# Rhodopsin gene expression regulated by the light dark cycle, light spectrum and light intensity in the dinoflagellate *Prorocentrum*

**DOI:** 10.3389/fmicb.2015.00555

**Published:** 2015-06-02

**Authors:** Xinguo Shi, Ling Li, Chentao Guo, Xin Lin, Meizhen Li, Senjie Lin

**Affiliations:** ^1^State Key Laboratory of Marine Environmental Science, College of Ocean and Earth Sciences, Xiamen UniversityXiamen, China; ^2^College of the Environment and Ecology, Xiamen UniversityXiamen, China; ^3^Department of Marine Sciences, University of ConnecticutGroton, CT, USA

**Keywords:** rhodopsin, gene expression, light dark cycle, light intensity, light spectrum, *Prorocentrum*

## Abstract

The proton pump rhodopsin is widely found in marine bacteria and archaea, where it functions to capture light energy and convert it to ATP. While found in several lineages of dinoflagellates, this gene has not been studied in Prorocentrales species and whether it functionally tunes to light spectra and intensities as in bacteria remains unclear. Here we identified and characterized this gene in the bloom-forming *Prorocentrum donghaiense*. It is a 7-helix transmembrane polypeptide containing conserved domains and critical amino acid residues of PPR. This gene is phylogenetically affiliated to the xanthorhodopsin clade, but seems to have a distinct evolutionary origin. Quantitative reverse transcription PCR showed that in regular cultures, the transcript abundance of the gene exhibited a clear diel pattern, high abundance in the light period and low in the dark. The same diel pattern was observed for protein abundance with a Western blot using specific antiserum. The rhythm was dampened when the cultures were shifted to continuous dark or light condition, suggesting that this gene is not under circadian clock control. Rhodopsin transcript and protein abundances varied with light intensity, both being highest at a moderate illumination level. Furthermore, the expression of this gene responded to different light spectra, with slightly higher transcript abundance under green than blue light, and lowest abundance under red light. Transformed *Escherichia coli* over-expressing this rhodopsin gene also exhibited an absorption maximum in the blue–green region with slightly higher absorption in the green. These rhodopsin-promoting light conditions are similar to the relatively turbid marine habitat where the species forms blooms, suggesting that this gene may function to compensate for the light-limited photosynthesis in the dim environment.

## Introduction

Microbial rhodopsin is a type of photoreceptor widespread in the marine ecosystem, widely reported in marine bacteria and archaea (de la Torre et al., 2003; Man et al., 2003; Venter et al., 2004; Giovannoni et al., 2005). It also occurs in some eukaryotic algae (Gualtieri et al., 1992; Nagel et al., 2002; Sineshchekov et al., 2005; Frassanito et al., 2010; Lin et al., 2010; Slamovits et al., 2011; Guo et al., 2014). Based on functional variations, rhodopsin can be classified as light-driven proton pumps, chloride pumps, Na^+^ pumps and signal transducers (Ernst et al., 2014). Among these functional types, the proton pump rhodopsin (PPR), in association with all-trans retinal, absorbs light and drives light-activated proton across cell membranes to generate an outward proton gradient. This results in proton outflux and production of ATP (Martinez et al., 2007). Thus PPR enables cells to acquire energy from light independently of plastid photosystems (Sharma et al., 2006; Walter et al., 2007; Gomez-Consarnau et al., 2010). Compared with the photochemical reaction in photosynthetic organisms, the rhodopsin-based phototrophic mechanism is more efficient due to the simplicity of the molecular machinery needed.

Proton pump rhodopsin was first discovered in archaea *Halobacterium salinarum* in early 1970s (Oesterhelt and Stoeckenius, 1971), and is now known to exist in marine γ-Proteobacteria (Beja et al., 2000) as well as a wide range of other bacteria (de la Torre et al., 2003; Venter et al., 2004). In recent years, PPR-like coding genes were found in some cultured and uncultured dinoflagellates, haptophytes, and diatoms (Lin et al., 2010; Marchetti et al., 2012). The detection of PPR genes in phylogenetically diverse taxa of dinoflagellates suggests that PPR occurs widely in this phylum (Lin et al., 2010). Yet, this gene has not been reported in the order of Prorocentrales, which are common in the world’s oceans.

Factors that regulate rhodopsin gene expression in eukaryotes remain poorly studied. Researchers have mainly examined the effect of nutrient and light conditions on PPR in bacteria. For example, it has been reported that in *Vibrio* strain AND4, rhodopsin gene expression was affected by nutrient limitation; in nutrient limited media, rhodopsin gene expression in this strain was strongly up-regulated leading to increased survival of the strain (Akram et al., 2013). Similar studies have been conducted for other bacteria to detect physiological functions of rhodopsin and their effects on population growth (Lami et al., 2009; Gomez-Consarnau et al., 2010; Steindler et al., 2011). These studies indicate that at least in some bacterial strains, rhodopsin enhances survival of the host species of bacteria under nutrient deficiency. Meanwhile, light has also been shown to influence rhodopsin gene expression in bacteria (Gomez-Consarnau et al., 2010). In *Dokdonia* sp. strain MED134, rhodopsin gene expression increased in light-cultivated cultures compared to dark-grown cultures (Gomez-Consarnau et al., 2010). In freshwater microbial communities, a metatranscriptome study showed that rhodopsin was expressed at a higher level in the light than in the dark (Vila-Costa et al., 2013). Furthermore, the rhodopsin gene also has been reported to respond to different spectra at different water depths. Shallow seawater tends to favor green-light-absorbing rhodopsin, while deeper waters favor blue-light-absorbing rhodopsin (Man et al., 2003; Fuhrman et al., 2008). The two types of rhodopsins in SAR86, with a single amino acid residue substitution at position 105, display different maximal absorbance spectra potentially enabling adaptation to their respective environments (Beja et al., 2001; Man et al., 2003).

In this study, we identified a rhodopsin gene from the Prorocentrales dinoflagellate *P. donghaiense*, which is a dominant harmful algal bloom (HAB) species in the East China Sea, where it forms HABs almost every year (Lu et al., 2005). As HABs formed by this species have been linked to relatively weak light field in the subsurface layer of a turbid water column (Sun et al., 2008), it is of interest to examine how the expression of this rhodopsin gene responds to changes in light conditions. We investigated the expression dynamics of *P. donghaiense* rhodopsin under different light/dark regimes, light intensities, and light spectra to deduce the potential contribution of this gene to enhanced fitness in *P. donghaiense*. To more closely link the gene transcriptional pattern to function, we also developed an antiserum and used it to determine *P. donghaiense* rhodopsin protein abundance in the cultures grown under different light intensities.

## Materials and Methods

### Algal Culture and Sample Collection for Rhodopsin Gene Identification

*Prorocentrum donghaiens* was originally isolated from a HAB event in East China Sea in 2009, and was provided by the Center for Collections of Marine Algae in Xiamen University (source culture number:CCMAXU-364). In this study, the culture was first grown in L1 medium with an antibiotic cocktail (200 mg/L ampicillin, 100 mg/L kanamycin and 100 mg/L streptomycin). The culture was verified to be bacteria free microscopically by DAPI staining (Life Technologies, Grand Island, NY, USA) of filtered samples and molecularly by 16S rDNA PCR of extracted DNA. To perform our experiments, the axenic culture of *P. donghaiense* was then transferred into fresh autoclaved L1 seawater medium (without silicate) at 20 ± 1°C under a 14:10 h light:dark cycle with a photon flux of 100 μE⋅m^-2^⋅s^-1^. The experiments were carried out in Yiheng incubators (Yiheng Technical Co., Ltd, China), with illumination provided by fluorescent light bulbs (Foshan Illumination Company, China). Cell counts were taken daily using a Sedgwick-Rafter counting chamber under the microscope, and growth curves were plotted to indicate growth stages. When the culture entered the mid exponential growth phase, cells (∼10^7^ cells per sample) were harvested by centrifugation at 3000 × *g* at 20°C for 10 min. The cell pellets were resuspended in 1 ml TRIzol Reagent (Invitrogen, Carlsbad, CA, USA) and stored at −80°C for subsequent RNA extraction.

### Light Manipulation to Study Rhodopsin Expression Pattern in *P. donghaiense*

The first experiment was carried out under a 14:10 h light dark regime. Culture was first synchronized as previously reported (Shi et al., 2013), and the synchronized culture was then transferred into 7.5-L L1 medium in triplicate. Three days later, when the cultures were in the exponential phase, 400 ml samples were collected as described above every 2 h over a 24-h light/dark cycle. Cell pellets were thoroughly resuspended in 1 ml TRIzol Reagent by vortex and stored at −80°C until RNA extractions.

In the second experiment, in order to measure rhodopsin expression levels under continuous light and continuous darkness, the synchronized culture grown as described above was split into two groups, with three replicates of each sample. One group was transferred to continuous illumination and the other group to continuous darkness. Twenty-four hours later, a sample was taken every 2 h for a 24 h period. This set of samples has been used previously on Rubisco gene expression (Shi et al., 2013).

In the third experiment, cultures were grown under different spectra to determine the response of *P. donghaiense* rhodopsin expression to chromatic variations. Triplicated cultures were grown under red (T8 30W/R, wavelength 622–700 nm, peak at 660 nm), green (T8 30W/G, wavelength 492–577 nm, peak at 560 nm) and blue lights (T8 30W/B, wavelength 455–492 nm, peak at 470 nm) provided by fluorescent lamps (Foshan Illumination Company, China) at equal intensities (100 μE⋅m^-2^⋅s^-1^) measured using digital luxmeter (TES1332A, Taiwan). Cell concentrations were determined daily as described earlier. From day 3 to day 7, samples were collected daily from each culture at the same time of the day using centrifugation as described above.

The fourth experiment was carried out to study *P. donghaiense* rhodopsin expression under different light intensities. A synchronized culture was split into three groups, with three replicates of each. The cultures were grown under 14:10 light dark cycle, at 100 (for convenience named normal light here), 20 (low light), and 200 μE⋅m^-2^⋅s^-1^ (high light). Daily sampling and cell concentration determination were performed as described above.

To further measure *P. donghaiense* rhodopsin protein abundance under different light intensities and light-dark cycle, the synchronized culture was split into groups (three replicates each) that were grown under four light intensities (25, 50, 100, and 200 μE⋅m^-2^⋅s^-1^). Cells were collected 4 h after the onset of the light period and 2 h after the onset of the dark period. Samples were harvested as described above. The cell pellets were resuspended in 0.5 ml PBS (phosphate-buffered saline) for subsequent protein extraction.

### RNA Extraction and cDNA Synthesis

Total RNA was extracted using TRI-Reagent (Molecular Research Center, Inc., Cincinnati, OH, USA) coupled with Qiagen RNeasy Mini kit (Qiagen) following previously reported protocol (Lin et al., 2010). The potential DNA contaminant was eliminated using RQ1 DNase (Promega) according to the manufacturer’s protocol and the resultant RNA was purified using Qiagen RNeasy Mini kit. RNA concentrations were measured using NanoDrop ND-2000 Spectrophotometer (Thermo Scientific), and the qualities were assessed using the absorbance ratios of 260/280 nm and 260/230 nm.

For each sample, 300 ng total RNA was used in cDNA synthesis. GeneRacer oligo-dT (Invitrogen, Carlsbad, CA, USA) was used as the primer in the case where the resultant cDNA was for rhodopsin gene amplification. For cDNA to be used in RT-qPCR, oligo-(dT)_16_ primer was used.

### Identification of *P. donghaiense* Rhodopsin cDNAs and Sequence Analysis

A rhodopsin gene fragment was obtained from a transcriptomic dataset generated using GS-FLX+ Titanium (454 Life Sciences, Roche, Branford, CT, USA) sequencing (unpublished data). A gene specific forward primer was designed based on the partial gene sequence (*PCDH-rhod-F*, **Table 1**) and was used with cDNA 3-end adaptor primer to amplify the 3′ end of the cDNA. PCR was run under the program consisting of initial denaturation at 95°C for 3 min, followed by 35 cycles 95°C 15 s, 56°C 30 s, 72°C 45 s, and a final step of 72°C for 5 min. The cDNA amplicon was purified, cloned, and sequenced. A gene specific reverse primer (*Pdrhod*-qR, **Table 1**) was paired with DinoSL as the forward primer (Lin et al., 2010) to amplify the 5′ end of the cDNA.

**Table 1:** Primers used in this study.

Primer name	Sequences (5′–3′)	Application	Source
DinoSL	TCCGTAGCCATTTTGGCTCAAG	dinoflagellate mRNA 5′- end cDNA synthesis and PCR (forward)	Zhang et al. (2007)
*PCDH-rhod-F*	GAGTCGRGCGCCTCWGAAGYYATGGTGA	*P. donghaiense* rhodopsin forward	This study
*Pdrhod*-QF	ATCCAGATCGGCTAYTGTGTCTC	*P. donghaiense* rhodopsin qPCR forward	This study
*Pdrhod*-QR	TTGGCATATGTGACCTGGTAGAT	*P. donghaiense* rhodopsin qPCR reverse	This study
Pdong-Cal-QF	AGTTCAAGGAGGCGTTCTCTTTGTTC	*P. donghaiense Calmodulin* qPCR forward	Shi et al. (2013)
Pdong-Cal-QR	CCATCAAGGACAAGAACTCGGGAAAG	*P. donghaiense Calmodulin* qPCR reverse	Shi et al. (2013)
Pdrhod-cF	ATGGTGATGTACCCGATGAGCGATA	*P. donghaiense* rhodopsin protein expression	This study
Pdrhod-cR	TCAAGCAAGCAGGGCCCCATCC	*P. donghaiense* rhodopsin protein expression	This study

Transmembrane helical structure was analyzed using ProteinPredict (Yachdav et al., 2014^1^). Protein secondary structure was displayed by web software TOPO2^2^. Multi-alignment was conducted to identify conserved amino acid residues that are presumed to form retinal pocket and those that are known to be functional residues in other species.

### Phylogenetic Analysis

To determine the affinity of the *P. donghaiense* rhodopsin, phylogenetic trees were constructed using the amino acid sequences of this gene. Reference protein sequences identified from BLAST results were retrieved from NCBI to combine with the sequences generated from this study. Alignment of these sequences was carried out using ClustalX (Larkin et al., 2007). Phylogenetic trees were inferred using neighbor-joining (NJ; Saitou and Nei, 1987) and Bayesian (BE; Huelsenbeck and Ronquist, 2001) methods. BE analysis was run for 100,000 generations with trees sampled every 100 cycle and the first 25,000 were discarded as burn-in. Tree topology was shown by the result of NJ analysis (with JTT amino acid substitution method) and support of the nodes was obtained from both BE and NJ analyses.

### Gene Expression Analysis Using Reverse Transcription Quantitative PCR (RT-qPCR)

Reverse transcription quantitative PCR was performed with cDNA templates prepared from samples collected from the various experiments described above, using iQTM SYBR^®^ Green Supermix in 96-well plates on a CFX96 Real-time PCR System (BioRad, USA). Each reaction was carried out in a total volume of 12 μl containing 250 nM of each primer, 5 μl cDNA or DNA, and 6 μl 2×SYBR^®^ Green Super mix. To prepare a standard curve, PCR amplicon of *P. donghaiense* rhodopsin was obtained from a plasmid containing the whole coding region. To achieve accurate standards, amplicon or restriction digested plasmid, instead of whole plasmid were used (Hou et al., 2010). The amplicon was purified and quantified using NanoDrop, and then serially diluted by 10-fold to obtain a gradient of 10^2^–10^7^ gene copies per 5 μl. The standard series and the experimental cDNA samples were run on the same PCR plates using the thermo cycle program as reported previously (Zhang and Lin, 2003). All reactions were carried out in three technical replicates. Data were analyzed using CFX software (Bio-Rad, Hercules, CA, USA). In order to normalize rhodopsin gene expression across different samples, several reference genes, including calmodulin (*calm*), glyceraldehyde 3-phosphate dehydrogenase (*gapdh*), a-tubulin and mitochondrial cytochrome b (*cob*), were selected to compare their expression stability (Supplementary Figure S3). *calm* showed the greatest stability and was selected to normalize expression levels of rhodopsin. The expression level of *calm* was also determined on the same qPCR plates as rhodopsin, with its standard curve (Supplementary Figure S4) prepared as previously reported (Shi et al., 2013).

### Rhodopsin Antibody Preparation and Western Blot Analysis

A synthetic peptide with the sequence CVTYAKSNKDGALLA, identical to the C terminus of the rhodopsin, was produced and used (peptide–KLH conjugate) to immunize two rabbits at Genscript Corporation (Piscataway, NJ, USA). The resultant polyclonal antibodies (PdRHODab1 and PdRHODab2) were affinity-purified and tested for titer using enzyme-linked immunosorbent assay. PdRHODab1, with a high titer (1:512,000), was chosen for use in this study.

Samples collected from the light dark regime and light intensity experiments were homogenized in PBS buffer as for RNA extraction. The homogenate was then centrifuged at 3000 × *g* at 4°C for 15 min, and the supernatant equivalent to 5 × 10^5^ cells from each sample was mixed with Laemmli buffer, and incubated at 95°C for 5 min. The samples were then loaded in 10% SDS-PAGE gels (Bio-Rad) and electrophoresed at 100 V for 1 h. The resolved proteins were transferred to a polyvinylidene difluoride (PVDF) membrane (Millipore, Bedford, MA, USA) using Trans-Blot SD Semi-Dry Transfer Cell (Bio-Rad, USA) at 25 v for 30 min. Membranes were blocked with 5% non-fat milk for 2 h and then incubated with the rhodopsin antiserum with 5000-fold dilution in PBS for 2 h at room temperature. Following three time washes with PBS containing 0.05% Tween 20 (PBST), the membrane was incubated with a biotinylated goat anti-rabbit IgG (TransGen Biotech, Beijing, China) in 10,000-fold dilution for 1 h at room temperature and then washed seven times in PBST. Finally, the membrane was washed and incubated with a horseradish peroxidase-labeled streptavidin solution (Beyotime Institute of Biotechnology, Shanghai, China). The immunoreactive bands were detected using the enhanced chemiluminescent (ECL) Substrate (Invitrogen, Carlsbad, CA, USA). The immunodetection procedure was essentially the same as we previously reported (Lin et al., 1994). The protein band image was captured using Bio-red Gel Doc XR. Following the same procedure, glutaraldehyde phosphate dehydrogenase (GAPDH; Ku et al., 2013) was detected on a protein blot prepared in parallel to the one used for rhodopsin protein detection. The primary antibody against GAPDH provided by Sangon (Cat #: AB90090, Shanghai, China) was used at 1:1000 dilution. Band intensity was measured using Bio-red Gel Doc XR equipped with Quantity One software (Bio-Rad Laboratories, ShangHai, China).

### Spectroscopy to Determine *P. donghaiense* Rhodopsin Absorption Optima

*Prorocentrum donghaiense* rhodopsin encoding sequence was amplified from the full-length rhodopsin cDNA using primer Pdrhod-cF and Pdrhod-cR (**Table 1**). The product was cloned into pEASY-E1 expression vector (TransGen Biotech) and transformed into *Escherichia coli* strain BL21. The *E. coli* culture was grown with all-*trans* retinol (final concentration 0.01 mM) and IPTG (final concentration 1 mM) to induce *P. donghaiense* rhodopsin expression for 3 h at 30°C with shaking at 200 rpm. Three miniliters of the culture were used to measure absorption spectrum in standard 1-cm cuvettes on Cary 100 spectrophotometer (Varian Instruments). Absorption was scanned from 450 nm to 650 nm at 0.1 nm intervals. Rhodopsin absorption spectrum was obtained by subtracting the spectrum of a negative control from the spectrum of the experimental culture; the negative control was *E. coli* strain BL21 transformed with the cloning vector without a target gene insert.

## Results

### *P. donghaiense* Rhodopsin Identification, Function Prediction and Phylogenetic Inference

A full-length cDNA (1013 bp, with spliced leader in the 5′-UTR and polyA in the 3′-UTR region was obtained (GenBank accession number, KM282617), and BLAST result showed that it was a rhodopsin. The cDNA encoded a protein of 258 amino acid residues with predicted molecular mass of 28.8 kDa. Transmembrane domain analysis predicted that *P. donghaiense* rhodopsin has seven transmembrane domains (Supplementary Figure S1A), the conserved feature of rhodopsin.

This gene also contains the same conserved functional residues as *Oxyrrhis marina* rhodopsin of the proton pump type (Slamovits et al., 2011). As shown in Supplementary Figure S1B, position 96 is an Asp (101 in *O. marina*), which is predicted to be a proton acceptor; position 107 (112 in *O. marina*) is Glu, a proton donor; and position 235 (237 in *O. marina*) is Lys, which is predicted to form the retinal pocket to harbor the retinal. There is a Leu residue at position 104, equivalent to position 105 in eBAC31A08 that has been shown to be a green-light-absorption-tuning switch residue (Man et al., 2003). Further, there is a Trp in position 155 (156 in *Salinibacter*), a hallmark of Xanthorhodopsin subgroup II, in contrast to Gly at this position in subgroup I (Vollmers et al., 2013). Substitution of Gly in this position by the bulky Trp abolishes binding of keto-carotenoids (Imasheva et al., 2009; Slamovits et al., 2011).

**FIGURE 1:**
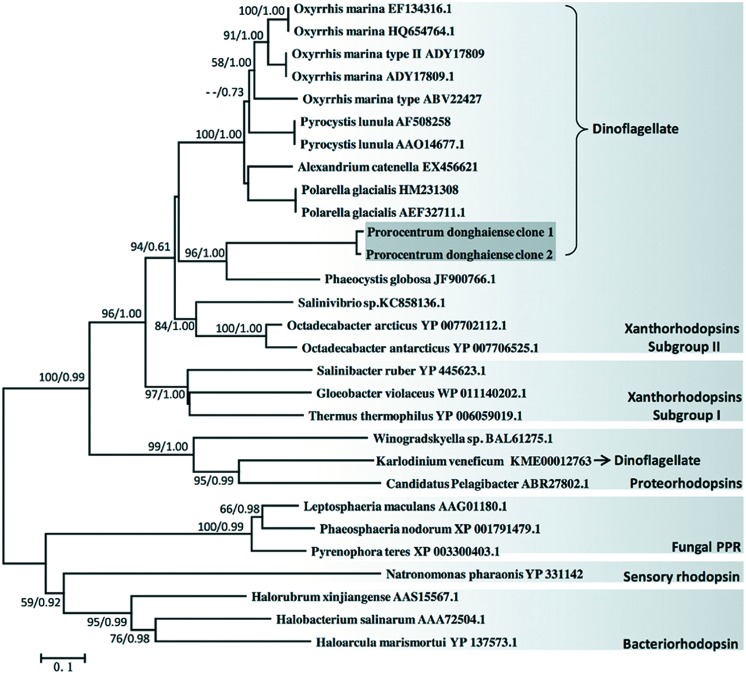
**Phylogenetic relationship of dinoflagellate rhodopsin with other typical rhodopsins based on amino acid sequences**. Tree topology shown is from neighbor-joining (NJ) analysis, which is similar to that produced by Bayesian (BE) analysis. Values shown at nodes are bootstrap support of NJ/posterior probability of BE analyses (only values >50%/0.50 are shown). General grouping of microbial rhodopsin is shown in separate light gray boxes with names placed on the lower right of the box. Dark gray box highlights *P. donghaiense* rhodopsin sequences obtained in this study. Bracket and arrow depict dinoflagellate proton pump rhodopsin (PPR) groups.

Phylogenetic analysis of amino acid sequences showed that *P. donghaiense* rhodopsin clustered with most of the dinoflagellate rhodopsins in a clade that otherwise consisted exclusively of rhodopsins from proteobacteria. This dinoflagellate rhodopsin clade belongs to xanthorhodopsin subgroup II (**Figure 1**). The only obvious exception was *Karlodnium veneficum* (formerly *K. micrum*), which was affiliated in a separate clade mainly composed of proteorhodopsins from bacteroidetes *Winogradskyella* and proteobacteria *Pelagibacter*. Additionally, we also found a very strong bootstrap support (100% from NJ and 0.99 from Mr. Bayes) for the affinity between the xanthorhodopsin clade and the proteorhodopsin clade. The apparent monophyletic grouping of major dinoflagellate rhodopsins was disrupted by rhodopsin recently reported from the haptophyte *Phaeocystis globosa* that branched with *P. donghaiense* rhodopsin into a distinct subclade, separated from the major dinoflagellate subclade. However, bootstrap support of the separation was not significant, probably due to too few taxa in the *Prorocentrum*/*Phaeocystis* cluter.

### *P. donghaiense* Rhodopsin Expression Profile Under LD, DD, and LL Light Regimes

**FIGURE 2:**
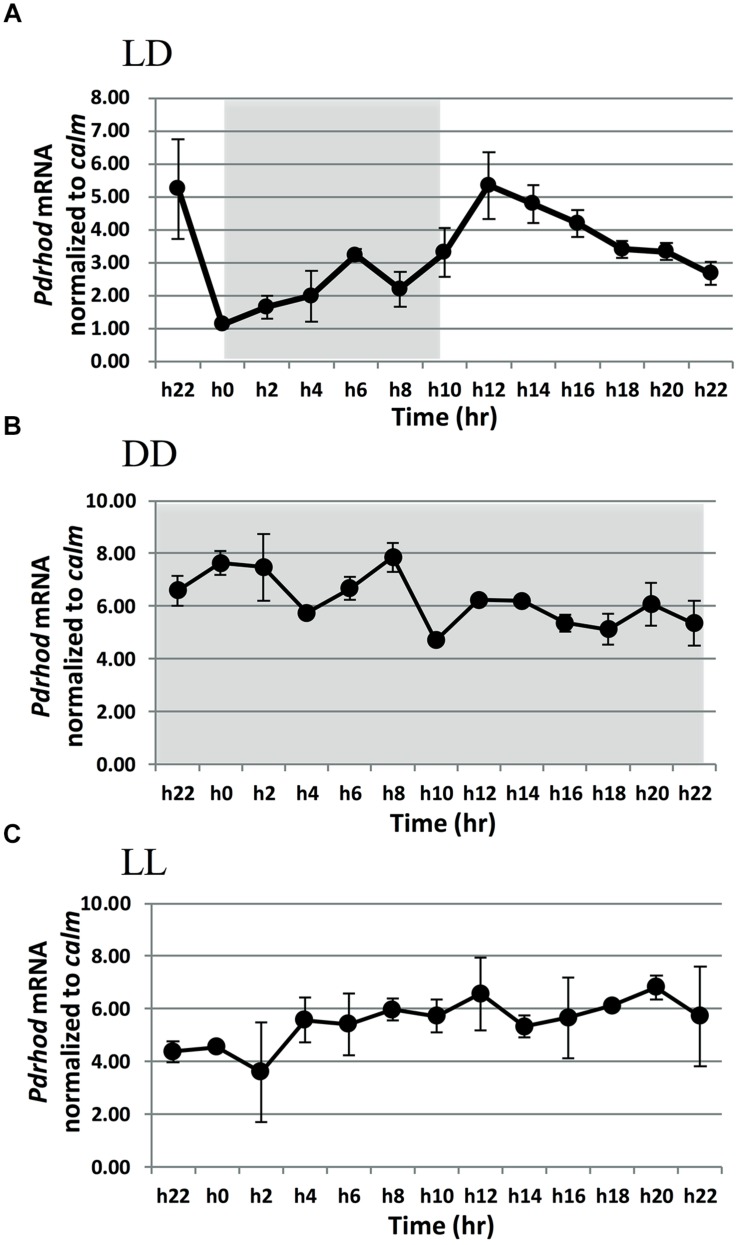
***Prorocentrum donghaiense* rhodopsin gene transcript dynamics normalized to calmodulin (*calm*; A,B,C) under different light dark regimes. (A)** LD: under light/dark cycle. **(B)** LL: under continuous light. **(C)** DD: under continuous darkness. Light gray shading denotes dark period. Error bars indicate ± SD of biological triplicates.

Rhodopsin transcript abundance relative to reference gene *calm*, exhibited a clear diel rhythm when the culture was cultured under the LD cycle with a photon flux of 100 μE⋅m^-2^⋅s^-1^ (**Figure 2A**). At the beginning of the dark period (h0), the transcripts abundance was in a lower level. It increased slowly from the middle (h4) to the late part of the dark period (h8). After the light period began (h10), *P. donghaiense* rhodopsin transcript abundance increased rapidly to reach a maximum in the middle of the light period (h12). Thereafter, the transcript level declined until the dark period. Throughout the LD cycle, the amplitude of the rhodopsin transcript dynamics was 4.8-fold. The same expression dynamic pattern was observed when rhodopsin transcript abundance was normalized to total RNA (Supplementary Figure S2A), except that the peak appeared 2 h later, and the amplitude of the dynamics was 6.5-fold.

In the DD and LL cultures, the expression level of *P. donghaiense* rhodopsin did not exhibit the same rhythm as that under LD (**Figures 2B,C**). The transcript abundance fluctuated only slightly, with a fold change of 1.7 and 1.6 in the DD cultures whereas 1.9 and 2.4 in the LL cultures when normalized to the reference genes *calm* and total RNA, respectively.

### *P. donghaiense* Rhodopsin Expression Profile Under Different Light Spectra and Intensities

**FIGURE 3:**
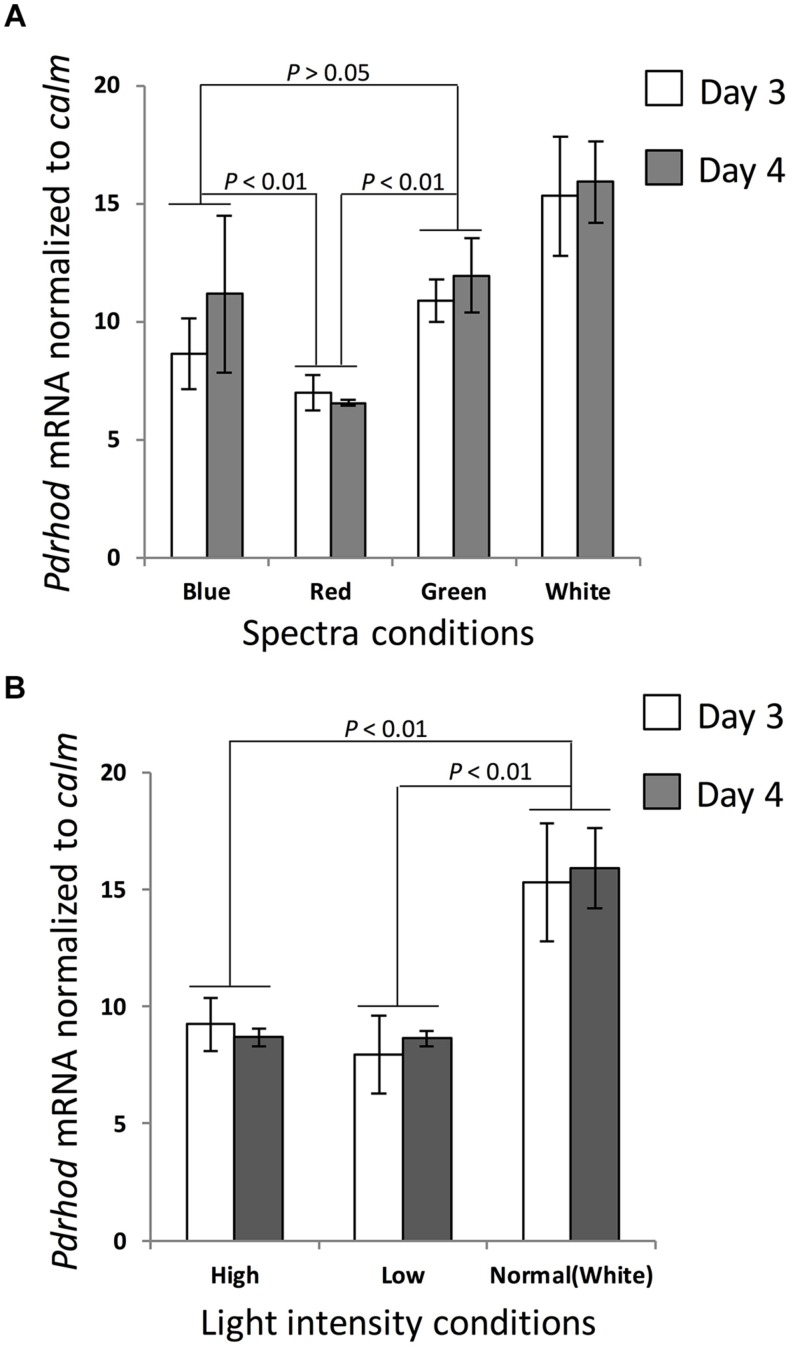
***Prorocentrum donghaiense* rhodopsin gene transcript dynamics under various light conditions**. Gene transcription level was normalized to calmodulin (*calm*). **(A)** Cultures under different spectra. **(B)** Cultures under different light intensities. Error bars indicate ± SD of biological triplicates. *p* values of pairwise comparison (*t*-test) are shown on each pair depicted by dotted lines.

Rhodopsin transcript abundance normalized to *calm* was significantly higher when the cultures were exposed to white, blue, and green light spectra than when exposed to red light (one-tailed *t-*test, *p* < 0.01, *n* = 6; **Figure 3A**, Supplementary Table S1). The average expression level under green and blue light was about 1.55-fold and 1.23-fold higher respectively than culture exposed to red light. Cultures exposed to green light showed a slightly higher expression level than exposed to blue light, but not significantly (one-tailed *t*-test, *n* = 6).

Under the “normal” photon flux (100 μE⋅m^-2^⋅s^-1^), rhodopsin transcript abundance maintained a relatively stable level (**Figure 3B**, Supplementary Table S2). In contrast, when the culture was shifted to a lower (20 μE⋅m^-2^⋅s^-1^) or a higher light intensity (200 μE⋅m^-2^⋅s^-1^), gene expression decreased markedly (one-tailed *t*-test, *p* < 0.01, *n* = 6), both to a relatively stable and similar level, more so when normalized to *calm* (**Figure 3B**).

### *P. donghaiense* Rhodopsin Protein Abundance Under Different Light Intensities and Light-Dark Cycle

**FIGURE 4:**
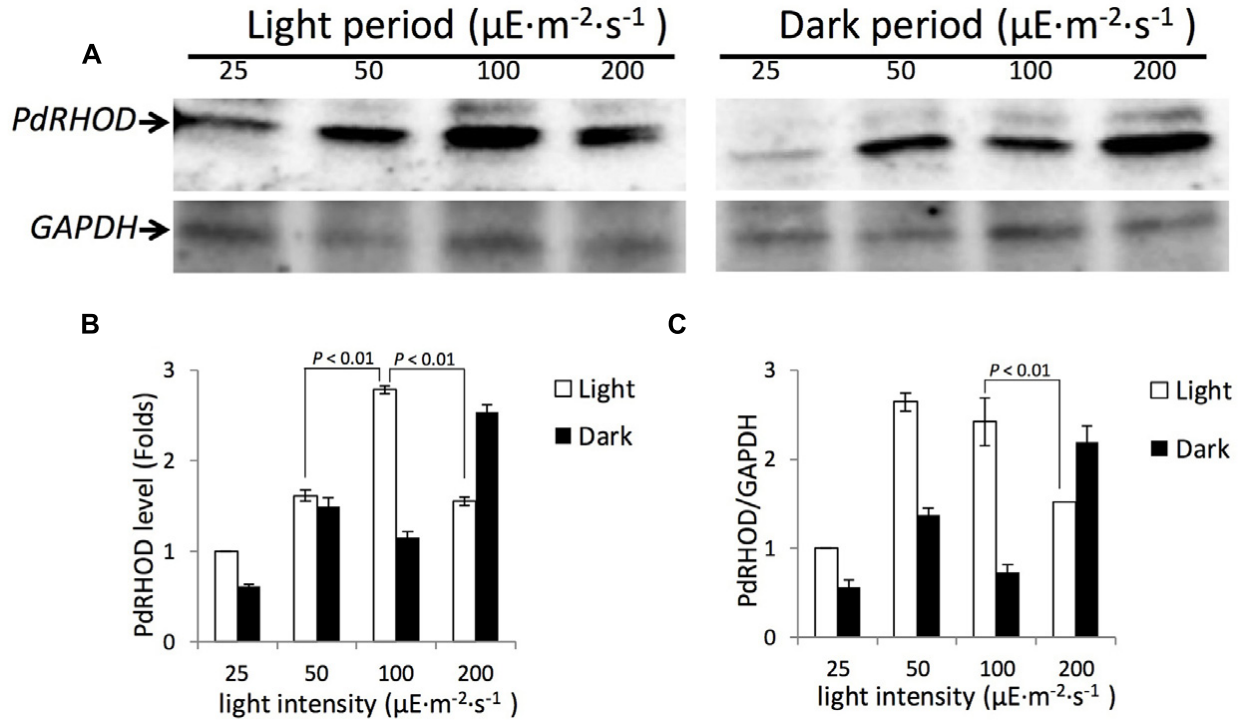
**Western blot analysis of *P. donghaiense* rhodopsin for cultures grown under different light densities. (A)** Immunoblot images of *P. donghaiense* rhodopsin and GAPDH for cultures grown under 25, 50, 100, and 200 μE⋅m^-2^⋅s^-1^. Analysis was done for both samples from light (left) and dark (right) periods. **(B)** Densitometric analysis of protein rhodopsin from the Western blot shown in **(A)**. The rhodopsin protein expression level is relative to that under light intensity of 25 μE⋅m^-2^⋅s^-1^ during the light period set as 1.0. Error bars indicate ± SD of biological triplicates. **(C)** Dynamics of rhodopsin normalized to GAPDH under different light densities during the light and dark periods. The rhodopsin to GAPDH ratios are relative values calculated by setting the abundance of both proteins under light intensity of 25 μE⋅m^-2^⋅s^-1^ during the light period as 1.0. Error bars indicate ± SD of biological triplicates. *p* values of pairwise comparison (*t*-test) are shown on solid lines.

*Prorocentrum donghaiense* rhodopsin was significantly more abundant in the light period than in the dark period, except when the culture was in 200 μE⋅m^-2^⋅s^-1^ (**Figure 4**). Under 25 μE⋅m^-2^⋅s^-1^, 50 μE⋅m^-2^⋅s^-1^, and 100 μE⋅m^-2^⋅s^-1^, the protein levels in the light period were 1.07–2.42 folds higher than in the dark period when normalized to the amount of total proteins and the fold change increased to 1.75–3.32 when normalized to the reference protein GAPDH. In the 200 μE⋅m^-2^⋅s^-1^ treated cultures, *P. donghaiense* rhodopsin was 1.63-folds higher in the dark than in the light period when normalized to the amount of total proteins, and the fold change was 1.44 when the expression level was normalized to GAPDH.

Meanwhile, *P. donghaiense* rhodopsin abundance in the light period showed a parabolic profile with light intensity. It was in a low level under 25 μE⋅m^-2^⋅s^-1^. The expression increased steadily with light intensity increase, reaching the highest level at 100 μE⋅m^-2^⋅s^-1^. At this light intensity level, the protein abundance was about 2.8-folds higher than that under 25 μE⋅m^-2^⋅s^-1^. Yet at the light intensity of 200 μE⋅m^-2^⋅s^-1^ the expression level decreased (**Figure 4**). When the protein level was normalized to GAPDH, a similar expression profile was detected, except for the abundance at 50 μE⋅m^-2^⋅s^-1^ being slightly higher than in 100 μE⋅m^-2^⋅s^-1^. The effect of light intensity seemed to extend to the dark period. Compared to the expression level in 25 μE⋅m^-2^⋅s^-1^, 2.3, 1.77 and 3.89-folds up regulation ware detected in 50 μE⋅m^-2^⋅s^-1^, 100 μE⋅m^-2^⋅s^-1^, and 200 μE⋅m^-2^⋅s^-1^ respectively (**Figure 4A**). The trend remained the same when the expression levels were normalized to GAPDH (**Figure 4C**).

### Blue-Green Absorption Spectrum of Over-Expressed *P. donghaiense* Rhodopsin

**FIGURE 5:**
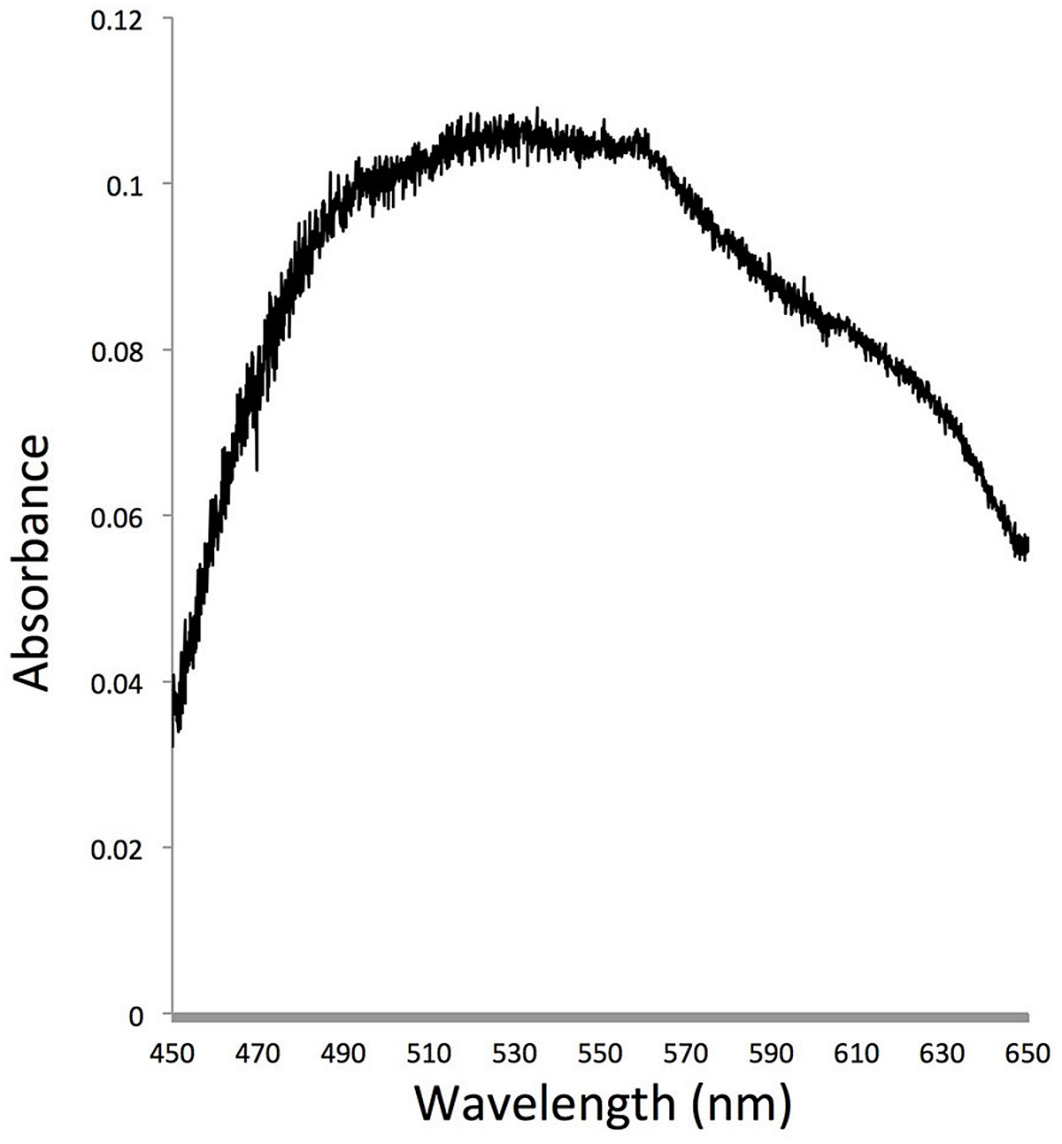
**Absorption spectrum of *E*. *coli* cells carrying *P. donghaiense* rhodopsin gene in an expression vector**. The maximum absorption of this protein located at 530 nm.

Spectroscopic analysis showed that the bacteria over-expressing *P. donghaiense* rhodopsin had a broad absorption peak in the blue-green spectrum, with green absorption slightly higher than blue absorption (**Figure 5**).

## Discussion

Photochemical reaction centers and retinal-activated proton pumps (PPR) are two different mechanisms used by organisms to harness solar energy. The former usually involves at least 30 plastid enzymes to form a complex system to harvest solar energy and fix carbon dioxide to provide energy for cell growth. The latter, in contrast, employs a simple mechanism to form a proton gradient to activate ATPase using a single membrane protein rhodopsin (Beja et al., 2001; Finkel et al., 2013), which is presumably more efficient. Therefore, it would seem favorable for an organism to harbor this light energy harvesting mechanism to supplement photosynthetic apparatus. That may explain why PPR is so widespread in the aquatic ecosystem (Venter et al., 2004). As an ecologically successful group of aquatic eukaryotic microbes, it is not surprising that PPR exists widely in dinoflagellates. The presence of the conserved critical residues (those making retinal pocket, electron donor and acceptor) and light-responding features observed in this and previous studies suggests that dinoflagellate rhodopsins of this kind (aside from the sensory type) likely have a similar function to bacterial PPR (Lin et al., 2010; Slamovits et al., 2011). As dinoflagellate PPRs are believed to have been acquired through horizontal gene transfer (HGT) from bacteria (see next section), functional conservation is expected. Both groups of organisms use PPR for the same reason. However, the functional extrapolation of bacterial PPR to dinoflagellate rhodopsin should be taken with caution due to the significant difference in cellular and molecular machinery between bacteria and eukaryotes. Direct experimental evidence, e.g., measured proton pump activity, is still required to verify the physiological function of rhodopsin in this species.

### The Affiliation of *P. donghaiense* Rhodopsin with Xanthorhodopsin Subgroup II and Evolution of Dinoflagellate Rhodopsins by Horizontal Gene Transfer

The presence of DinoSL at the 5′end of *P. donghaiense* rhodopsin cDNA indicates that the sequence was indeed from a dinoflagellate rather than from bacteria. All dinoflagellate rhodopsins except some in *O. marina* (which possesses both sensory and proton pump types of rhodopsin) belong to PPR type. Within this type, there is a xanthorhodpsin group, which is further divided into subgroups I and II (Vollmers et al., 2013). Our phylogenetic inference clearly placed *P. donghaiense* rhodopsin, along with all other dinoflagellate PPRs (except those in *K. veneficum*), in xanthorhodopsin subgroup II. This affiliation has strong statistical support (96% NJ bootstrap/1.00 BE posterior probability). This suggests that dinoflagellate rhodopsins do not bind to the 4-keto-carotenoid antenna pigments (Vollmers et al., 2013).

The separation of *K. veneficum* rhodopsin from typical dinoflagellate rhodopsins in our phylogenetic tree agrees with previous findings and lending further support to the proposition that dinoflagellate rhodopsins have arisen at least twice independently through HGT (Lin et al., 2010; Slamovits et al., 2011). Furthermore, our observation that *P. donghaiense* rhodopsin branched with a haptophyte rhodopsin in a distinct subclade suggests that *P. donghaiense* rhodopsin might have been acquired in yet another HGT event, and shares with the haptophyte *Phaeocystis* a common bacterial rhodopsin progenitor. This requires more rigorous phylogenetic analysis in the future with broader taxon sampling to achieve significant bootstrap support. Whereas *K. veneficum* plastid is haptophyte originated (Tengs et al., 2000), it is curious that *K. veneficum* rhodopsin is so distantly separated from haptophyte rhodopsin. However, this is not entirely surprising given that rhodopsin and chloroplast have independent evolutionary histories.

### The Expression of Rhodopsin is Light Dark Cycle-Dependent in *P. donghaiense*

Understanding how the expression of rhodopsin responds to illumination variability sheds light on the protein’s function in the organism. Both rhodopsin transcript and protein in *P. donghaiense* showed higher abundances in the light period under normal light intensity (100 μE⋅m^-2^⋅s^-1^) than in the dark. Our qPCR result showed a remarkable diel oscillation in rhodopsin gene transcription: low transcript abundances in the dark with a minimum in the mid-dark period, and high transcript abundances in the light period with a peak in the mid-light period. The same diel rhodopsin expression pattern also has been detected in a meta-transcriptomics study on phosphorus limited lake microbial community, where the dominant type of rhodopsin was bacterial PPR (Vila-Costa et al., 2013). Similarly, rhodopsin protein abundance in the light period was significantly higher than in the dark period when the cells were cultured under normal light intensity (100 μE⋅m^-2^⋅s^-1^), as shown in our Western blot result. All the data suggest that rhodopsin expression may be light-dark cycle related.

The observed diel rhythm may be attributed to a circadian clock or simply to a light/dark-triggered oscillation. Circadian clock regulated rhythm would persist at least 2–3 days after the shifting from light dark (LD) cycle to continuous light (LL) or continuous darkness (DD) (Hwang et al., 1996). Therefore, we transferred the LD-grown culture to LL and DD, respectively, and analyzed rhodopsin transcript abundance over a 24 h period. The transcript abundance under LL and DD did not display the diel rhythm that was observed under LD. This result suggests that rhodopsin expression is directly influenced by light dark conditions, and is not under circadian clock control. This illumination-responsive feature would enable the organism to promptly tune to varying light conditions in its habitat.

It is interesting to note that the transcript abundance of *P. donghaiense* rhodopsin in LL and DD were basically the same in our study. This is different from rhodopsin gene expression profile in bacteria such as Flavobacteria and SAR11 (Gomez-Consarnau et al., 2007; Lami et al., 2009), in which the abundance of rhodopsin transcripts was dramatically higher under LL or LD than under DD (Lami et al., 2009). While PPR depression in DD seen in bacteria is as what would be expected for a light absorbing protein, the lack of difference between DD and LL in *P. donghaiense* cannot be explained. There is a possibility that the expression of this gene is controlled by light dark transition cues as previously proposed for phytoplankton (Fuhrman et al., 2008). This requires further investigation.

### Effects of Spectrum and Light Intensity: Potentially Adaptive to Natural Light Field in *P. donghaiense* Habitat

Cruise surveys and field experiments of *P. donghaiense* in East China Sea have revealed that the highest cell density occurred at middle water depths (deep chlorophyll maximum layer, usually located at 10–50 m in *P. donghaiense* bloom area) prior to a bloom outbreak (Sun et al., 2008; Chen et al., 2011; Wen et al., 2012). At this depth, long wavelength spectra such as red and yellow would have largely disappeared due to absorption by particles and water molecules, leaving green and blue light as the major available spectra (Kirk, 1994). Our qPCR results of samples collected from cultures grown under different spectra suggest that the rhodopsin transcript level under green light was somewhat higher than it was under blue light, but both were significantly higher (by 1.67 ± 0.41 folds when data from all sampling days were considered and 1.83 ± 0.30 folds if the last day data were excluded) than under red light (*p* < 0.05). In accordance, *P. donghaiense* rhodopsin is presumably a green-light-absorbing type rhodopsin because of the Leu residue at the position 104 (equivalent to position 105 in eBAC31A08). PPR with Leu at this position has been shown to have an absorption maximum in the green light spectrum in bacteria such as SAR86 and *Dokdonia sp.* strain MED134 (Man et al., 2003; Gomez-Consarnau et al., 2007), which is believed to promote bacterial growth under green light condition. Furthermore, the absorption spectrum from *P. donghaiense* rhodopsin cloned into and over-expressed in *E. coli* also showed the maximum absorption in the green spectrum although it has a broad absorption peak from blue to green. As chloroplast mainly absorbs blue light, green light dominates coastal waters, making the green shift of absorption spectrum highly adaptive in the coastal marine environment (Beja et al., 2000).

Previous reports have suggested that light intensity is one of the most important factors influencing the bloom dynamics of *P. donghaiense* in East China Sea (Chen et al., 2006). This species normally blooms at relatively muddy sea areas where light penetration is relatively low (∼175 ± 17.4 μE⋅m^-2^⋅s^-1^; Sun et al., 2008). As PPR can putatively function as a source of energy subsidy, it is of interest to examine how the expression of this protein/gene responds to different light intensities. When the cultures were treated with different light intensities, the transcript abundance of *P. donghaiense* rhodopsin showed the highest level in cells grown under a moderate light intensity (100 μE⋅m^-2^⋅s^-1^) and decreased considerably when the cultures were transferred to low (20 μE⋅m^-2^⋅s^-1^) or high (200 μE⋅m^-2^⋅s^-1^) light conditions. The same expression pattern was detected in two consecutive days. Our Western blot results also showed that the encoded protein too was most abundant at moderate to lower light intensities (100 μE⋅m^-2^⋅s^-1^ when it was normalized to total protein, 50 μE⋅m^-2^⋅s^-1^ when normalized to reference GAPDH). Thus, both the qPCR and the Western blot data in concert suggest an adaptation of the PPR system in *P. donghaiense* to this organism’s commonly occurring turbid habitat. The decrease in both transcript and protein abundances of *P. donghaiense* rhodopsin at 200 μE⋅m^-2^⋅s^-1^ perhaps should not be a surprise, in light of a previous study on bacterium *Psychoflexus torquis* showing higher PPR expression levels under dim light than under high light or darkness (Feng et al., 2013). The authors in that study suggested that *P. torquis* PPR expression under high light was influenced by photooxidative stress, because the bacteria cell abundance and growth rate was lower under this illumination condition. However, it seems unlikely in our case, because both cell abundance and growth rate were not decreased under 200 μE⋅m^-2^⋅s^-1^, at which photooxidative stress was suggested not to be so likely to take place in *P. donghaiense* (Xu et al., 2010). Based on current data, it is not clear why *P. donghaiense* rhodopsin transcript and protein abundance decreased under high light.

In summary, our qRT-PCR and Western blot results showed that *P. donghaiense* rhodopsin expression profile (high expression in the medium light intensity, during light periods, and under green/blue light wavelength) implies that this chloroplast-independent light energy harvesting system will enhance fitness of this organism under bloom conditions. These are consistent with what would be expected of a functional PPR. The light intensity as well as chromatic optima of rhodopsin *in P. donghaiense* expression is likely a consequence of evolutionary adaptation to the organism’s living environment, including subsurface layer of a turbid water column. Therefore, this protein may provide *P. donghaiense* a fitness advantage, allowing it to outgrow other phytoplankton, which rely on chloroplast light harvesting system, and form intense blooms in turbid subsurface seawater. Even in calm clear water column, PPR with the light responding features of this protein will allow a dinoflagellate (able to migrate with the aid of its flagella) to photosynthetically utilize the more abundant nutrients at the nutricline depth even though light is dim there. However, further experiments, such as measure proton pump activity of *P. donghaiense* rhodopsin using techniques such as laser flash photolysis and gene knockout, are needed to prove this hypothesis.

## Conflict of Interest Statement

The authors declare that the research was conducted in the absence of any commercial or financial relationships that could be construed as a potential conflict of interest.

## Footnotes

1 www.predictprotein.org

2 http://www.sacs.ucsf.edu/cgi-bin/open-topo2.py
