# Supplementary material for: Rhodopsin gene expression regulated by the light dark cycle, light spectrum and light intensity in the dinoflagellate Prorocentrum
Source: Front Microbiol. 2015 Jun 2;6:555. doi: 10.3389/fmicb.2015.00555 (PMC4451421; doi:10.3389/fmicb.2015.00555)
Supplement: Supplementary file 1 [file Table_1.DOC]

Table S1. Differential expression analysis of *Pdrhod* (normalized to *calm*) in cultures cultivated under red, green, blue and white light conditions.

| Spectra | Day 3 (Average±SD) | Day 4 (Average±SD) | *t*-test (*p*-value) | | |
| --- | --- | --- | --- | --- | --- |
|  |  |  | blue_VS_red | green_VS_red | blue_VS_green |
| blue | 8.63±1.49 | 11.17±3.32 | 0.0098  (Days 3 and 4 combined, n=6) | 5.04E-06  (Days 3 and 4 combined, n=6) | 0.12  (Days 3 and 4 combined, n=6) |
| red | 7.01±0.75 | 6.56±0.14 |  |  |  |
| green | 10.87±0.90 | 11.96±1.55 |  |  |  |
| white | 15.31±2.53 | 15.91±1.73 |  |  |  |
