# Supplementary material for: Rhodopsin gene expression regulated by the light dark cycle, light spectrum and light intensity in the dinoflagellate Prorocentrum
Source: Front Microbiol. 2015 Jun 2;6:555. doi: 10.3389/fmicb.2015.00555 (PMC4451421; doi:10.3389/fmicb.2015.00555)
Supplement: Supplementary file 2 [file Table_2.DOC]

Table S2. Differential expression analysis of *Pdrhod* (normalized to *calm*) in cultures cultivated under low, high and normal light conditions

| Condition | Day 3 (Average±SD) | Day 4 (Average±SD) | *t*-test (*p*-value) | |
| --- | --- | --- | --- | --- |
|  |  |  | High light VS Normal | Low light VS Normal |
| High light | 9.23±1.13 | 8.69±0.38 | 8.46E-06  (Days 3 and 4 combined, n=6) | 6.48E-06  (Days 3 and 4 combined, n=6) |
| Low light | 7.95±1.66 | 8.63±0.33 |  |  |
| Normal | 15.31±2.53 | 15.91±1.73 |  |  |
