# Supplementary material for: Rhodopsin gene expression regulated by the light dark cycle, light spectrum and light intensity in the dinoflagellate Prorocentrum
Source: Front Microbiol. 2015 Jun 2;6:555. doi: 10.3389/fmicb.2015.00555 (PMC4451421; doi:10.3389/fmicb.2015.00555)
Supplement: Supplementary file 3 [file Image_1.PDF]

Figure S1. Secondary structure of *P. donghaiense* rhodopsin protein and alignment with homologs from *Oxyrrhis marina* and bacteria

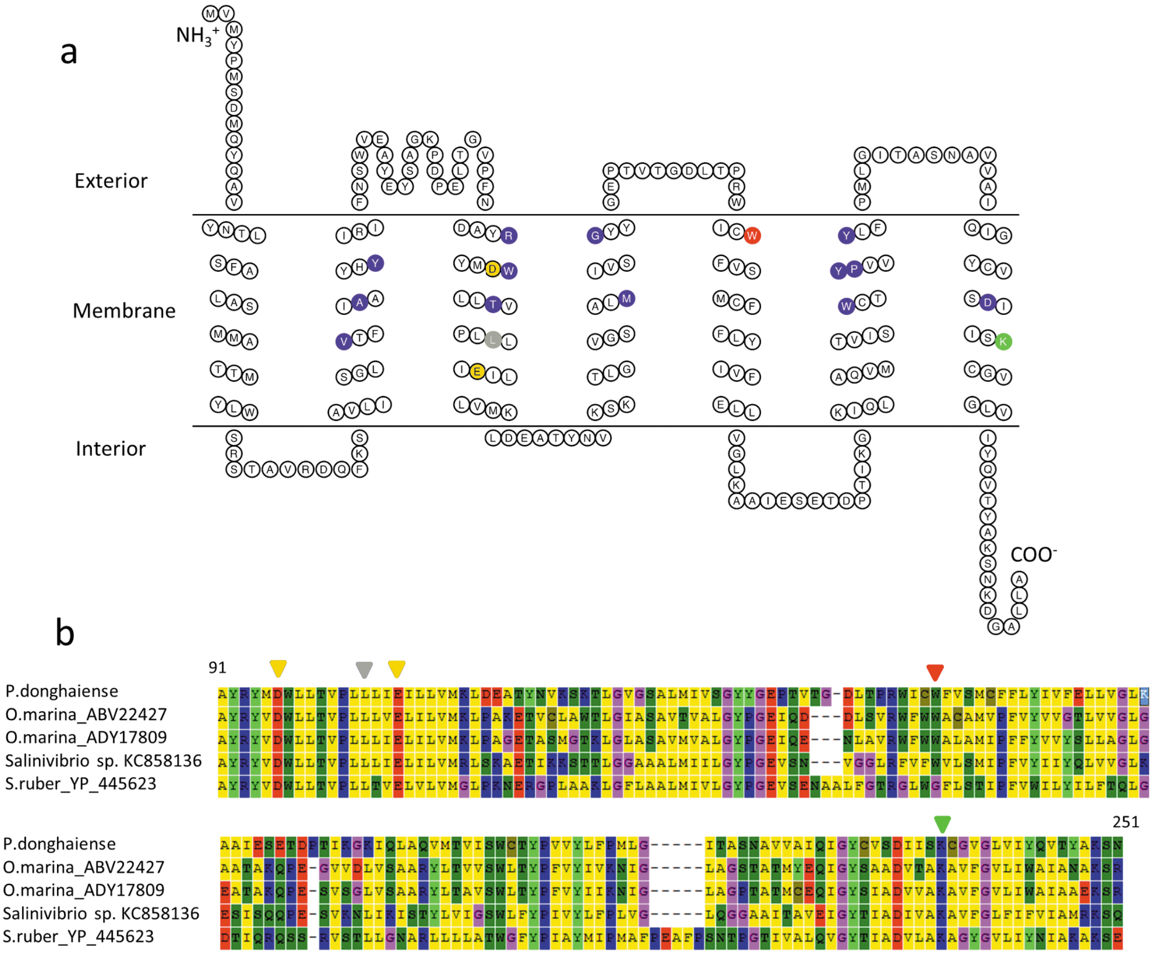

Figure S1. Secondary structure of *P. donghaiense* rhodopsin protein (a) and alignment with homologs from *Oxyrrhis marina* and bacteria (b). (a) Secondary structure predicted using ProteinPredict. Functional residues are highlighted in different colors: purple, retinal binding pocket; yellow, proton acceptor (residue D) and donor (residue E); grey, spectral tuning; red, keto-carotenoids binding; green, lysine linked to the cofactor retinal. (b) Residues 91-251 region is shown the functional sites marked with triangles. Same color triangles as in (a) are used to depict conserved functional sites. O.marina_ABV22427, *O. marina* type I rhodopsin; O.marina_ADY17809, *O. marina* type II rhodopsin; *Salinivibrio* sp. KC858136, Xanthorhodopsins Subgroup II; *S.ruber*_YP_445623, Xanthorhodopsins Subgroup I rhodopsin; Position 96 and position 107 (marked with black triangle) function as proton acceptor and donor respectively; position 104 (marked with blue triangle) function as spectral tuning; position 155 (marked with red triangle) is the residue critical for keto-carotenoids binding; position 235 is critical for forming the retinal pocket to link the retinal.
