# Supplementary material for: Rhodopsin gene expression regulated by the light dark cycle, light spectrum and light intensity in the dinoflagellate Prorocentrum
Source: Front Microbiol. 2015 Jun 2;6:555. doi: 10.3389/fmicb.2015.00555 (PMC4451421; doi:10.3389/fmicb.2015.00555)
Supplement: Supplementary file 4 [file Image_2.PDF]

Figure S2. *P. donghaiense* rhodopsin gene transcription dynamics normalized to total RNA under different light dark regimes

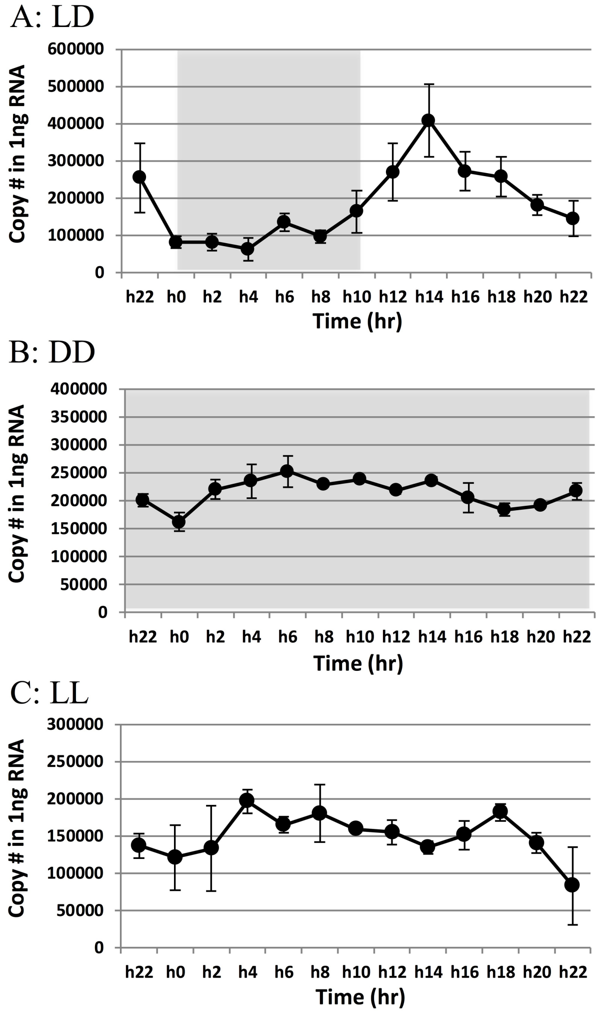

Figure S2. *P. donghaiense* rhodopsin gene transcription dynamics normalized to total RNA under different light dark regimes. LD: under light/dark cycle. LL: under continuous light. DD: under continuous darkness. Light grey shading denotes dark period. Error bars indicate ± standard deviation of biological triplicates
