# Supplementary material for: Rhodopsin gene expression regulated by the light dark cycle, light spectrum and light intensity in the dinoflagellate Prorocentrum
Source: Front Microbiol. 2015 Jun 2;6:555. doi: 10.3389/fmicb.2015.00555 (PMC4451421; doi:10.3389/fmicb.2015.00555)
Supplement: Supplementary file 5 [file Image_3.PDF]

Figure S3. Reference gene stability compare

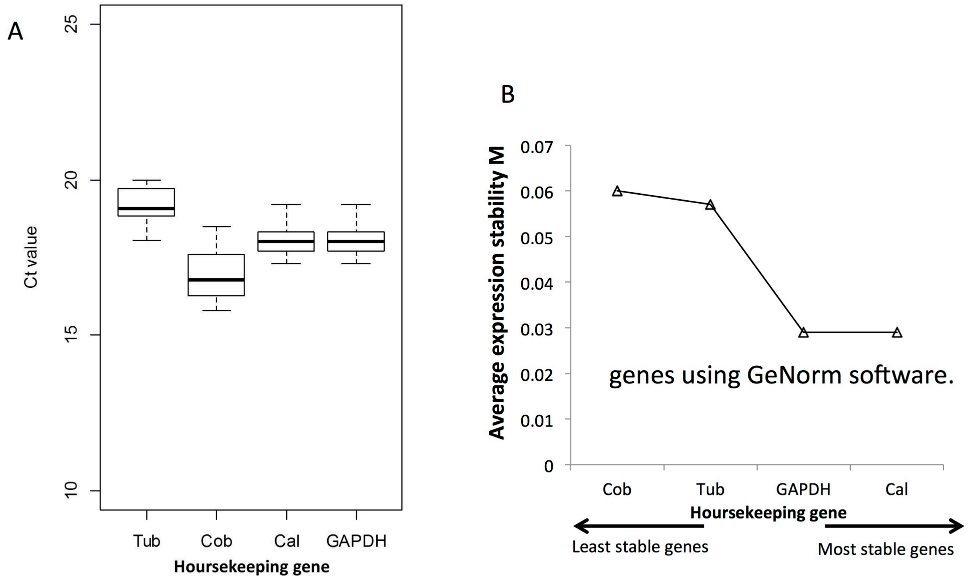

Figure S3. Reference gene stability compare. (A) Range of real-time PCR cycle threshold (*C*T) values of 4 HKGs in samples. *C*T variability data in HKG comparisons are shown as median (lines), 25–75% (boxes), and range (whiskers) for all sample pools. (B) The analysis of average expression stability (M) of potential reference genes using GeNorm software.
