# Supplementary material for: Rhodopsin gene expression regulated by the light dark cycle, light spectrum and light intensity in the dinoflagellate Prorocentrum
Source: Front Microbiol. 2015 Jun 2;6:555. doi: 10.3389/fmicb.2015.00555 (PMC4451421; doi:10.3389/fmicb.2015.00555)
Supplement: Supplementary file 6 [file Image_4.PDF]

Figure S4. Standard curve for qPCR.

A

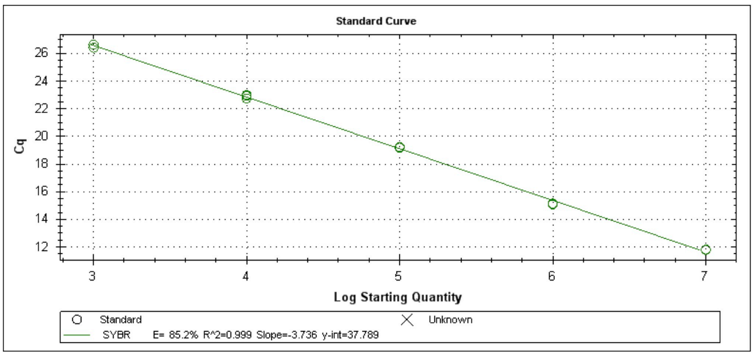

B

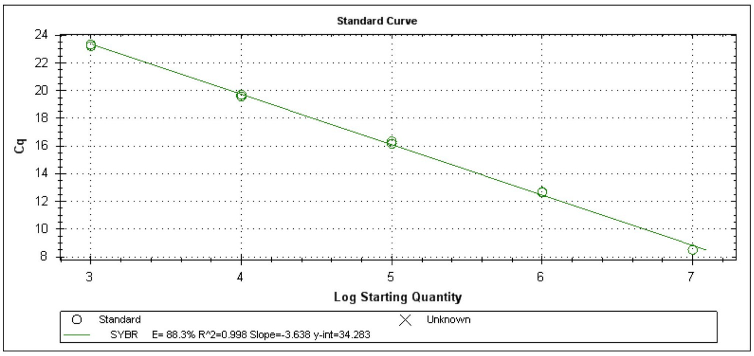

Figure S4. Standard curve for qPCR. (A) the standard curve of calm. (B) the standard curve of rhodopsin.
